# Using a commercially available DNA extraction kit to obtain high quality human genomic DNA suitable for PCR and genotyping from 11-year-old saliva saturated cotton spit wads

**DOI:** 10.1186/1756-0500-1-133

**Published:** 2008-12-22

**Authors:** Erik A Ehli, Timea Lengyel-Nelson, James J Hudziak, Gareth E Davies

**Affiliations:** 1 Avera Institute for Human Behavioral Genetics, Avera Behavioral Health Center, Sioux Falls, SD, USA; 2 University of Vermont, College of Medicine, Burlington, VT, USA; 3 South Dakota State University, College of Pharmacy, Brookings, SD, USA; 4 University of South Dakota, Sanford School of Medicine, Department of Psychiatry, Sioux Falls, SD, USA

## Abstract

**Background:**

We sought to describe the integrity of human genomic DNA extracted from saliva saturated cotton spit wads stored at -20°C for approximately 11 years. 783 spit wad samples were collected from an ADHD sample population (Vermont Family Study) during 1996–2000. Human genomic DNA was extracted from the spit wads using a commercially available kit; QIAamp DNA Blood Midi Kit (Qiagen, Inc., Valencia, CA.) with a few modifications.

**Results:**

The resulting DNA yield was more than adequate for genetic analysis and ranged from approximately 1 μg to a total of 80 μg (mean 17.3 μgs ± 11.9 μgs). A_260_/A_280 _ratios for the human genomic DNA extracted from the spit wads was consistently within the generally acceptable values of 1.7–2.0, with the lowest purity being 1.70, and a mean value of 1.937 ± 0.226 for the 783 samples. The DNA also was suitable for PCR reactions as evidenced by the amplification of the serotonin-transporter-linked polymorphic region, 5HTTLPR. 5HTTLPR is a functional polymorphism in the promoter region of the serotonin transporter gene (*HTT, SLC6A4*, or *SERT*), consisting of two intensively studied alleles. 770 of the 783 samples (98.3%) produced fragments after PCR of the expected size with primers specific for 5HTTLPR.

**Conclusion:**

High quality and abundant genomic DNA can be successfully retrieved from saliva saturated cotton spit wads using the commercially available kit, QIAamp DNA Blood Midi Kit from Qiagen, Inc. Furthermore, the DNA can be extracted in less than 3 hours and multiple samples can be processed simultaneously thus reducing processing time.

## Background

Given the increasing emphasis the study of molecular genetic influences on the development of psychiatric disorders; simple, noninvasive and cost-effective methods of collecting DNA for large-scale studies are needed for real time and remote (after years of storage) genetic analyses. Whole blood, serum, and plasma have long been the gold standard for obtaining high quality, abundant genomic DNA suitable for genetics studies; however, research has shown that a blood draw may be a significant barrier for study participation, especially those studies involving pediatric patients [1] with complex psychiatric disorders such as attention deficit hyperactivity disorder (ADHD) and autism spectrum illness. Buccal cells have proven to be an effective painless procedure as a means to DNA collection from large sample populations [2]. In addition, the procedure is relatively quick, cost effective, and a non-invasive means to collect genomic DNA. Buccal cells can be collected using a variety of different methods including cytobrushes, clean sterile swabs, mouthwash, saliva alone, and in the case of the Vermont Family Study with spit wads.

Genetic analysis experiments require the genomic DNA from the study sample to be of adequate quantity and quality. The affymetrix 6.0 chips, which enable genotyping of up to 1.8 million genetic markers, requires 500 ng of total genomic DNA [3]. In addition, PCR reactions required to amplify microsatellite markers in candidate gene and linkage studies typically require 50 ng or more of genomic DNA per marker [4,5]. The quality of genomic DNA extracted for experiments are typically measured using spectrophotometric absorbance ratios of 260 nm/280 nm. High quality DNA is considered to have an A_260_/A_280 _ratio of 1.7–2.0. The quality of genomic DNA can also be measured using PCR success [6].

We sought to describe the integrity of DNA from saliva saturated cotton spit wads collected from 1996–2000 as part of an ADHD sample population known as the Vermont Family Study. The spit wads were stored at -20°C for 11 years and revisited at the present time to undergo various genetic experiments, including genotyping known candidate genes, whole genome association/methylation studies, and copy number variation experiments. The information from this study may be useful to anyone who has collected buccal cells for DNA isolation using a non-conventional method.

## Results

### DNA Yield

The quantity of genomic DNA extracted from the saliva saturated cotton spit wads was determined using the conventional method of absorbance at 260 nm (A_260_). We found the DNA yield from the 783 DNA extractions to be highly variable from sample to sample, with an average yield of 17.3 μgs with a standard deviation of ± 11.9 μgs. Genomic DNA yield ranged from 1 μg to as high as 80 μgs. By our own empirical observation, DNA yield seem to correlate with the saturation level of the spit wad. Spit wads heavily saturated with saliva generally produced a higher genomic DNA yield in comparison with drier spit wads.

### DNA Quality

The quality of DNA was assessed using two different methods. Initially, DNA quality was determined using an A_260_/A_280 _ratio. Additionally, because the main purpose for collecting the DNA was for future genotyping studies, we assessed DNA quality using PCR amplification of the 5HTTLPR polymorphism. The generally used convention of assessing DNA quality with an A_260_/A_280 _ratio is that pure genomic DNA will have a ratio between 1.7 and 2.0. The mean A_260_/A_280 _ratio for the 783 samples was determined to be 1.937 ± 0.226. Interestingly, samples concentrated using Microcon YM-100 centrifugal filter devices generally increased in purity as measured using the A_260_/A_280 _ratio. As a result, the adjusted mean A_260_/A_280 _ratio of the sample population after concentrating samples that fell below our selected 50 ng/ul cutoff was 1.965 ± 0.124.

**Figure 1:**
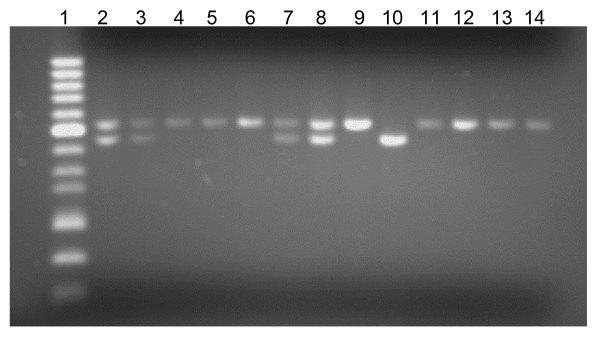
**Representative agarose gel of 5HTTLPR PCR fragments from Genomic DNA**. Representative 2% agarose gel showing fragments obtained from a PCR using genomic DNA isolated from Vermont Family Study spit wads and primers specific for 5HTTLPR polymorphism. Lane 1 contains 50 bp DNA ladder (Fisher Bioreagents), with a bold reference band of 400 bp. Lane 2 is a positive control sample with known S/L genotype. Lanes 3–14 show fragments generated from 12 samples using genomic DNA extracted from spit wads as template for 5HTTLPR PCR reaction. 12.5 μl of the 25 μl PCR reaction was mixed with 2 ul of 6X agarose DNA loading dye (Fisher Bioreagents) prior to loading the gel.

Using PCR analysis of the 5HTTLPR polymorphism we identified that 770 DNA samples (98.3%) were successfully amplified and detected utilizing fragment analysis on the ABI 3130 Genetic Analyzer. 48 DNA samples were randomly selected and PCR was repeated under the same conditions and fragments were identified as concordant using gel electrophoresis. A representative gel is shown in figure 1.

## Discussion

This methodology report describes the effectiveness of a commercially available kit in extracting and purifying human genomic DNA from saliva saturated cotton spit wads. We found that DNA obtained from the spit wads was of adequate quantity and quality for use in downstream genetic studies.

The yield of genomic DNA from buccal cells from spit wads was highly variable, with yields ranging from 1 μg to as high as 80 μgs. The variability observed with DNA yield is consistent with results from other studies using buccal cytobrushes in which yields are reported from 0.5 μgs to 12.66 μgs [8,6,10]. In our sample of 783 individuals, we generated a mean of 17.3 μgs ± 11.9 μgs of genomic DNA from the spit wads. The yield of genomic DNA achieved from the spit wads would be more than adequate for numerous genetics studies including microsatellite analysis, whole genome association studies, linkage analysis, copy number variation experiments, etc.

We also report an estimate of DNA quality extracted from the Vermont Family Study samples using an A_260_/A_280 _ratio to identify DNA purity and protein contamination. DNA is generally considered to be of adequate quality when the A_260_/A_280 _ratio is between 1.70 and 2.0. The quality of genomic DNA from the saliva saturated cotton spit wads, assessed using spectrophotometer readings, was more than adequate as the mean A_260_/A_280 _ratio for the 783 samples was 1.937 ± 0.226. Since the DNA was to be banked for future genetic studies, we imposed a 50 ng/μl concentration cut-off for each sample prior to being stored at -20°C. Samples with concentrations below this threshold were concentrated using Microcon YM-100 centrifugal filter units. Interestingly, after concentrating these samples through the filter unit, the purity as measured by an A_260_/A_280 _ratio increased. The adjusted mean after concentration of samples falling below the 50 ng/μl cutoff was 1.965 ± 0.124. Most likely, any contaminants or impurities are removed during the concentration procedure increasing purity of the genomic DNA prep.

In addition, the quality of genomic DNA was also assessed by PCR success using primers specific for the 5HTTLPR polymorphism. We identified a 98.3% success rate (770/783) with the genomic DNA as a template for this PCR reaction. A representative gel is shown in figure 1 showing the fragments observed from a PCR reaction with 5HTTLPR specific primers.

## Conclusion

This report describes a novel, cost effective, and efficient way to collect genomic DNA from individuals enrolling in genetic studies. This method should enable researchers to obtain high quality and abundant genomic DNA that can be successfully retrieved from saliva saturated cotton spit wads using the commercially available kit, QIAamp DNA Blood Midi Kit from Qiagen, Inc. Furthermore, the DNA can be extracted in less than 3 hours and multiple samples can be processed simultaneously thus reducing processing time and cost.

## Methods

### Participants

The Vermont Family Study is a collection of samples from 207 families comprising 783 individuals. 167 families were part of an ADHD sample with one member of each family recognized as a Proband for the disorder. 40 families were included as control families with no DSM-IV diagnoses.

### Sample Collection

Participants were asked to refrain from eating or drinking 1 hour prior to saliva collection. Each individual was instructed to place a standard 2" × 2" piece of cotton gauze in the buccal region of their cheek for 3 minutes. The saliva saturated cotton spit wad was removed, rolled to fit a collection tube, and stored at -20°C until the genomic DNA was extracted. All data collected and analyzed with approval of the UVM COM IRB Ethics Committee.

### DNA Extraction and Quantification

All DNA extractions were performed at the Avera Institute for Human Behavioral Genetics. DNA was extracted from saliva saturated cotton spit wads using a column-based purification method. Rolled spit wads measured approximately, 5 cm × 1.5 cm, and DNA was extracted from buccal cells using the QIAamp DNA Blood Midi Kit large volume protocol (Qiagen) according to the manufacturer's instructions with a few modifications. The spit wad was incubated at 70°C in a Protease/Lysis buffer mixture (200 ul Qiagen Protease/2.4 ml buffer AL) for 30 minutes in a 15 ml conical tube (Fisher Scientific). The lysate was separated from the spit wad using centrifugal force by placing the spit wad in the barrel of a 5 ml syringe (Becton Dickson) that was seated in a conical 15 ml tube and centrifuged for 10 minutes at 12,000 rpm. The spit wad was discarded and 2 ml absolute ethanol was added to the lysate and the tube was mixed by vigorous shaking (vortexing). Approximately one half of the lysate/ethanol mixture was transferred to a Qiagen Midi column placed in a clean 15 ml conical tube. The column was centrifuged @ 1850 × g for 3 minutes. The filtrate was discarded and the remaining lysate/ethanol mixture was applied to the same column and centrifuged @ 1850 × g for 3 minutes. The column was washed with 2 mls of buffer AW1 and centrifuged @ 3220 × g for 2 minutes. The column was washed a second time with 2 mls of buffer AW2 and centrifuged @ 3220 × g for 30 minutes to ensure complete drying. DNA was eluted from the column into a clean 15 ml conical tube by adding 200 μl buffer AE to the column, incubating at room temperature for 5 minutes, and centrifuging @ 3220 × g for 4 minutes. For maximum DNA yield a second elution was performed as described above, yielding approximately 400 μl total volume. DNA concentration and purity were determined using UV spectrophotometry (Nanodrop). All genomic DNA was either diluted or concentrated to a final concentration of 50 ng/ul. DNA was diluted in a reduced EDTA buffer (10 mM Tris-HCL, 0.1 mM EDTA, pH 8.0) and concentrated using Microcon YM-100 centrifugal filter devices (Millipore) according to the manufacturer's instructions.

### PCR Amplification

The Quality of DNA isolated from the spit wad was assessed by PCR amplification of the serotonin-transporter-linked polymorphic region (5HTTLPR). PCR products were visualized using 2% agarose gel electrophoresis or fragment analysis on an ABI 3130 Genetic Analyzer (Applied Biosystems, Inc.). Primer sequences for 5-HTTLPR were previously described; forward primer (5' -ATGCCAGCACCTAACCCCTAATGT-3') and the reverse primer (5'- GGACCGCAAGGTGGGCGGGA-3') [7]. When running samples on the 3130 genetic analyzer a fluorescently tagged forward primer (6FAM, Applied Biosystems) was used to tag the PCR product for fragment analysis. This primer pair amplifies a 419 base pair product for the 16-repeat long (L) allele and a 375 base pair product for the 14-repeat short (S) allele. PCR reactions were performed using a PCR Master Mix (Promega) containing a final concentration of 1.5 mM MgCl_2_, 1× reaction buffer, 200 μM of each dNTP, 40 ng purified genomic DNA, 1.25 units *Taq *DNA polymerase, and 5 pmols of each primer in a 25 ul reaction. PCR cycling conditions consisted of an initial denaturation at 95°C for 15 minutes, 35 cycles each consisting of 30 s at 94°C, 30 s at 66°C, and 40 s at 72°C. Elongation was continued for 15 min at 72°C after the last cycle. S vs. L fragments were called using GeneMapper Software Version 4.0 (ABI), or fragments were separated on a 2% agarose gel supplemented with ethidium bromide (0.02%, Fisher).

## Competing interests

The authors declare that they have no competing interests.

## Authors' contributions

EAE: Participated in design of study, manuscript preparation, DNA extraction, and PCR analysis, TLN: Participated in DNA extraction and PCR analysis, JJH: Designed and executed collection of spit wads in Vermont Family Study and manuscript preparation, GED: Participated in design of study and manuscript preparation
